# Sialylation-related gene signature predict prognosis and immunotherapy efficacy in low- and high-grade glioma: a PCA-based stratification study

**DOI:** 10.3389/fonc.2025.1676103

**Published:** 2025-12-09

**Authors:** Guidong Zhu, Yan Liu, Rui Liu, Guoqiang Xin, Chengke Zhang, Chengwei Wang

**Affiliations:** 1 Department of Neurosurgery, The Second Qilu Hospital of Shandong University, Jinan, China; 2 The Third Affiliated Hospital of Shandong First Medical University, Affiliated Hospital of Shandong Academy of Medical Sciences, Jinan, China; 3 Department of Oncology, Shandong Provincial Key Medical and Health Discipline, Qingdao Central Hospital, University of Health and Rehabilitation Sciences, Qingdao, China; 4 Department of Neurology, Sunshine Union Hospital of Shandong Province, Weifang, China; 5 Department of Thoracic Surgery, The Second Qilu Hospital of Shandong University, Jinan, China

**Keywords:** sialylation, tumor immune microenvironment, immunotherapy, predict prognosis, glioblastoma

## Abstract

**Background:**

Sialylation is a crucial glycosylation modification of eukaryotic cell surface proteins. Tumor cell growth, immune evasion, and drug resistance are driven by excessive sialylation. However, the expression levels of genes associated with sialylation, prognostic value, and impact on the response to immunotherapy in brain tumors remain unclear. This study hypothesized that sialylation-related genes could serve as grouping genes to identify 20 significant genes for predicting the survival outcomes of patients with brain tumors and their responsiveness to immune checkpoint inhibitors.

**Methods:**

Using 83 genes related to sialylation, we classified the cohort into two distinct groups with marked differences in survival outcomes and immune cell infiltration. After identifying the differential genes, we subsequently performed unsupervised clustering, yielding two groups with high concordance. Principal component analysis (PCA) was used for dimensionality reduction in the two groups, with the PC1 and PC2 components defined as the PCA score.

**Results:**

A positive correlation was observed between the PCA score and immune cell infiltration, immune checkpoint expression, as well as chemokine expression, suggesting enhanced immunotherapy efficacy. We also validated the expression levels of KCNIP3 in patient tissues and found that this gene was overexpressed in U87 and LN229 cells, with low endogenous expression. These results demonstrate that KCNIP3 inhibits tumor cell proliferation, migration, and invasion. Moreover, intracranial xenograft results in mice were consistent with the *in vitro* findings.

**Conclusion:**

We developed a robust model to predict patient prognosis in low-grade gliomas and glioblastomas, emphasizing the prognostic significance of differentially expressed genes associated with sialylation.

## Background

Gliomas are the most common malignant tumors of the central nervous system in adults (1), with an annual incidence of approximately 6.4 per 100,000 people (2). Gliomas are classified into four grades: grades 1 and 2 gliomas are referred to as low-grade gliomas, and grades 3 and 4 gliomas are high-grade gliomas (3). The prognosis of low-grade gliomas varies depending on the subtype, but is generally favorable, with a five-year survival rate exceeding 80%, although most patients eventually experience progression (4). High-grade gliomas, particularly glioblastomas (GBM), have an incidence rate of approximately 4.03 per 100,000 individuals, accounting for 50.1% of all cases. Even with standard multimodal treatments involving surgical resection, concurrent radiotherapy, and temozolomide (TMZ) chemotherapy, patient survival remains poor, with the median overall survival (OS) of patients with GBM improving from only 12 to 16 months (5). Immunotherapy offers hope for brain tumors treatment (6–9). However, as gliomas are among the “coldest” tumors immunologically, with a highly immunosuppressive tumor microenvironment (TME) and significant tumor heterogeneity (10), these factors often lead to the failure of immunotherapy (6). Therefore, the discovery of novel molecular targets and the development of new immunotherapeutic strategies hold promise for future brain tumor treatments.

Sialylation is a post-translational protein modification (11, 12). This process plays a critical biological role in maintaining cell-cell interactions and is associated with various pathological conditions including cancer, embryonic lethality, and immune system disorders (13). Previous studies have demonstrated that the upregulation of sialyltransferases and an abnormal increase in sialylation on the surface of tumor cells can directly influence cancer cell adhesion, motility, and invasiveness (14), thereby promoting tumor growth and metastasis (15, 16). Sialylation is regulated by sialyltransferases, transporters and members of the neuraminidase family (17). The human STs family comprises of 20 conserved enzymes that transfer sialic acid from CMP-Neu5Ac to the terminal glycans of various glycolipids and glycoproteins (18). By masking tumor antigen epitopes and promoting an immunosuppressive microenvironment, excessive sialylation on the surface of tumor cells allows cancer cells to escape immune surveillance (19, 20). As a result, sialyltransferases have recently been identified as promising targets for cancer treatment (21). Thus, we hypothesized that sialylation-related genes could serve as promising molecular biomarkers for predicting patient survival and immunotherapy efficacy.

We performed unsupervised clustering using 83 sialylation-related genes and identified two distinct groups within the cohort, showing marked differences in survival and immune infiltration. In this study, the unsupervised clustering analysis was performed on an integrated cohort comprising both low-grade glioma (LGG) and GBM patients. After identifying the differential genes between the two groups, unsupervised clustering was subsequently performed, yielding two groups with high concordance. Dimensionality reduction was performed using PCA in the two groups, and the PC1-PC2 components were defined as the PCA score. The score showed a positive relationship with immune infiltration, checkpoint expression, and chemokine production, implying better efficacy of immunotherapy. We further validated these findings in melanoma and non-small cell lung cancer cohorts. Our results demonstrated that this score was associated with the clinicopathological features, somatic mutations, and immune microenvironment of patients with brain cancer. We used this score to screen and verify the biological effects of KCNIP3 in gliomas, both *in vitro* and *in vivo*. This score could serve as a novel biomarker for estimating the mortality risk, suggesting that sialylation-related gene sets may contribute to personalized immunotherapy in patients with brain cancer.

## Methods

### Data source, preprocessing and human tumor sample collection

A total of 670 cases of LGG and GBM, including RNA transcriptome data and clinicopathological features, were retrieved from TCGA dataset via UCSC Xena (https://xenabrowser.net/datapages/). We also downloaded two batches of transcriptomic data from the CGGA database (https://cgga.org.cn/download.jsp): one containing 693 patient samples and the other containing 325 patient samples. Both datasets contained comprehensive clinical information. Bulk RNA-seq datasets were generated from the samples, and batch effects were corrected before merging the data using the R packages ‘limma’ and ‘sva’ (22).

GSCA (https://guolab.wchscu.cn/GSCA/#/) is used for GBM and LGG gene set analysis at the genomic levels. Mutation analysis of the 11 genes in GBM and LGG was performed using GSCA to calculate the correlations between CNV, SNV, and various tumors.

Between 2020 and 2022, a total of 20 human glioma tissue samples were collected from surgical resections of glioma patients at the Second Hospital of Shandong University. These 20 clinical samples were used for immunofluorescence staining to validate the expression of key sialylation-related prognostic genes in glioma tissues, which aimed to verify the consistency between the gene expression patterns observed in public transcriptomic datasets and the protein expression levels in clinical specimens, thus providing clinical evidence for the bioinformatics findings.

### Sialylation-related genes definition

The latest sialylation-related genes were identified using the Molecular Signatures Database (https://www.gsea-msigdb.org/gsea/msigdb).

### Differential analysis and functional enrichment analysis

Differential gene expression analysis was conducted to identify differentially expressed genes (DEGs) between the high- and low-expression groups, using the criteria |logFC| > 2 and FDR < 0.05.

To explore the biological functions of molecular subgroup-specific genes, Gene Ontology (GO) and Kyoto Encyclopedia of Genes and Genomes (KEGG) enrichment analyses were performed using the “clusterProfiler” R package based on DEGs. The gene set variation analysis (GSVA) algorithm calculates enrichment scores for specified gene sets in each sample. The normalized enrichment scores (NES) for each member of the reactome gene sets were computed using the GSVA method with the “GSVA” package in the LGG and GBM cohorts.

### Unsupervised consensus clustering

Univariate Cox regression revealed significant sialylation-related genes (p < 0.05). Kaplan-Meier survival curves were generated, and log-rank tests compared OS rates across these genes. The Univariate Cox Regression analysis in our study was based on OS. Using R’s ConsensusClusterPlus package [23], we performed consensus clustering with k-means (Euclidean distance; max k=9), where the optimal cluster number was selected based on PCA scores.

### Construction of a prognostic model and PCA score definition

We defined the PCA score of the individual samples as follows: PCA score = PC1–PC2.

### Tumor microenvironment and immune infiltration analysis

Gene expression data were analyzed using the ESTIMATE algorithm to quantify stromal and immune cell populations in tumor tissues, generating stromal, immune, and combined ESTIMATE scores for each sample. Immune infiltration patterns and their association with gene expression were subsequently evaluated through ssGSEA using the GSVA package, with Spearman’s correlation method (significance threshold: P < 0.05).

### Cell cultures

We cultured HEK293T, LN229, and U87 cells (Chinese Academy of Sciences Cell Bank, Shanghai) in DMEM (Gibco, USA) with 10% FBS and 100 U/ml penicillin-streptomycin at 37 °C/5% CO_2_. All cell lines underwent mycoplasma testing with negative results.

### Reagents

P Puromycin (Sparkjade Biotechnology Co., Ltd., Shandong, China) was prepared as an aqueous stock solution and stored at -20°C. Antibodies targeting β-actin (cat. no. YT0099) and GAPDH (cat. no. YT5052) were purchased from ImmunoWay Biotechnology (Plano, TX, USA), while the KChIP3 antibody (cat. no. PA5-99454) was acquired from Thermo Fisher Scientific, Inc. (Waltham, MA, USA).

### Immunofluorescence staining

Immunofluorescence staining was performed on the aforementioned 20 human glioma tissue samples to detect the protein expression of target genes, following the standard protocol below: (1) Deparaffinization and rehydration of tissue sections, (2) Antigen retrieval, (3) Blocking with 10% normal goat serum, (4) Primary antibody incubation (4 °C, overnight), (5) FITC-488-conjugated goat anti-rabbit secondary antibody application, followed by counterstaining with 4’,6-diamidino-2-phenylindole (DAPI) and mounting of sections, and (6) Images were captured using an inverted fluorescence microscope (Nikon, Japan), and the data were analyzed with ImageJ software.

### Plasmids construction, lentivirus packaging and transfection

The KCNIP3 DNA fragment was inserted into the pHBLV-CMV-MCS-EF1-Puromycin vector via HB-infusionTM (HanBio Biotechnology, Shanghai) following manufacturer protocols. HEK293T cells were co-transfected with either the recombinant plasmid (pHBLV-KCNIP3) or empty vector control, along with packaging plasmids pSPAX2 and pMD2G. Lentiviral supernatants were harvested 48–72 hours post-transfection and cryopreserved at -80°C. U87 and LN229 cells were transduced using viral supernatant with polybrene (10 μg/ml), followed by 14-day selection with puromycin (4 μg/ml).

### Cell proliferation assay

Cell viability was assessed using the Cell Counting Kit-8 (CCK-8; Sparkjade Biotechnology Co., Ltd., Shandong, China). Briefly, 8 × 10³ cells per well were seeded in 96-well plates and cultured at 37°C for 48 or 72 hours. Subsequently, 10 μL of CCK-8 reagent was added to 100 μL of serum-free DMEM in each well, followed by incubation at 37°C for 4 hours. Absorbance was measured at 450 nm using a Bio-Rad Model 680 microplate reader (Hercules, CA, USA).

### Wound healing assay

A standardized wound healing assay was performed by seeding LN229-KCNIP3 or U87-KCNIP3 cells (5 × 10^5^ cells/well) in pre-marked six-well plates. Upon reaching 80% confluence, uniform wounds were created using 200 μL pipette tips, followed by replacement with serum-free medium. Cell migration was monitored at 37°C/5% CO_2_, with images captured at 0, 6, 12, and 24-hour intervals using an inverted microscope. Wound closure quantification was performed using ImageJ software.

### Transwell assay

The Transwell invasion assay was performed by coating chambers with Matrigel diluted 1:8 in DMEM (60 μL/well) at 4 °C, followed by polymerization at 37 °C for 2 hours. LN229-KCNIP3 or U87-KCNIP3 cells (2 × 10^4^ cells in 200 μL serum-free DMEM) were seeded in the upper chamber, while the lower chamber contained 500 μL DMEM with 10% FBS as chemoattractant. After 24-hour incubation, invaded cells were fixed with 4% paraformaldehyde (15 min), stained with 0.1% crystal violet (30 min), and quantified using ImageJ software following microscopic visualization.

### *In situ* xenograft animal model

Male NOD mice (6–8 weeks old) were obtained from GemPharmatech Co., Ltd. (China) and maintained in SPF conditions. Animals were anesthetized with isoflurane (RWD Life Science, China; 2–3% induction, 1–2% maintenance) in an induction chamber, then immobilized in a stereotactic frame for intracranial injection of 5×10^5^ LN229-KCNIP3-luc cells (5 μL suspension) at 1.5 mm anterior to bregma, 2 mm lateral to midline, and 3 mm depth. Tumor progression was assessed biweekly using an IVIS imaging system (PerkinElmer, USA), with data quantification performed using Living Image 4.5.2 software (PerkinElmer, USA).

### Statistical analysis

R software version 4.4.1 was used for all bioinformatics statistical analyses. The R packages utilized in this study, including “clusterProfiler” (23), “sva”, “limma” (22), “ggplot2”, “ggpubr” (24), “survival”, and “survminer” (25), along with other relevant R packages, were obtained from the Bioconductor repository or the Comprehensive R Archive Network (CRAN). We presented all data as mean ± standard deviation and performed statistical analyses using GraphPad Prism 9.4.1 (La Jolla, CA, USA). Comparisons between two groups employed a two-tailed Student’s t-test, while one-way ANOVA analyzed multi-group data. The significance threshold was set at p < 0.05 (*P < 0.05, **P < 0.01, ***P < 0.001).

## Results

### Identification of two subtypes using unsupervised hierarchical cluster analysis based on a panel of sialylation-related genes

Before performing unsupervised clustering on the 109 sialylation-related genes (**Table 1**), three datasets (CGA325, CGAA693, and TCGA) were merged, and batch effects were corrected, followed by PCA for dimensionality reduction. The dimensionality reduction results of the three transcriptomic datasets are illustrated in **Supplementary Figures S1A, B**. After univariate Cox regression analysis, 83 genes with p < 0.05 were identified, and the top 20 genes were selected for correlation analysis (**Supplementary Figure S1C**). The results showed that Genes with HR < 1 included GALNT9, ST8SIA3, NEU4, GALNT13, ST6GAL2, RTN4R, and ST6GALNAC1, whereas those with HR > 1 included NANP, GLB1, CTSA, GALNT2, HEXB, GNS, ST8SIA4, CHST2, GALNT3, SIGLEC9, SIGLEC7, B3GNT7, and GALNT5.

**Table 1:** Sialylation-related genes.

Gene Symbol
ST3GAL1
ST3GAL2
ST6GALNAC1
ST8SIA3
ST8SIA4
ST8SIA2
ST3GAL3
ST6GALNAC3
ST6GALNAC4
ST6GALNAC6
ST8SIA6
ST6GALNAC5
ST3GAL5
NEU3
NEU4
NEU1
NEU2
ST3GAL4
ST3GAL6
ST6GALNAC2
ST6GAL1
ST8SIA5
ST8SIA1
ST6GAL2
GLB1
SLC17A5
GNE
SLC35A1
NANP
NANS
CTSA
CMAS
NPL
B4GALT5
GALNT6
GALNT5
GALNT13
GALNT15
GALNTL5
B3GNT6
GALNT1
GALNT2
GALNT3
GALNT8
GCNT1
B3GNT3
B3GNT2
B4GAT1
SLC35D2
CHST5
GNS
CHST6
B3GNT4
CHST1
B4GALT4
C1GALT1C1
GALNT18
GALNTL6
GALNT9
GCNT4
GALNT7
GALNT10
C1GALT1
GALNT16
GALNT11
GALNT17
GALNT14
GALNT12
GALNT4
GCNT3
C20orf173
B4GALNT1
B3GALT4
B4GALT6
GM2A
HEXA
HEXB
ABCA2
ITGB8
CLN6
MAG
SIGLEC14
SIGLEC11
FCN1
SIGLEC7
SIGLEC9
SIGLEC8
SIGLECL1
AGRN
SIGLEC16
SELE
SELP
SIGLEC5
SIGLEC10
SIGLEC12
CD22
ADIPOQ
CD33
SIGLEC6
B3GNT7
CHST2
SLC17A4
SLC17A2
SLC17A3
SLC17A1
CLIP3
EPDR1
PSAP
RTN4R

The 109 sialylation-related genes were identified by univariate Cox regression analysis.

**Figure 1:**
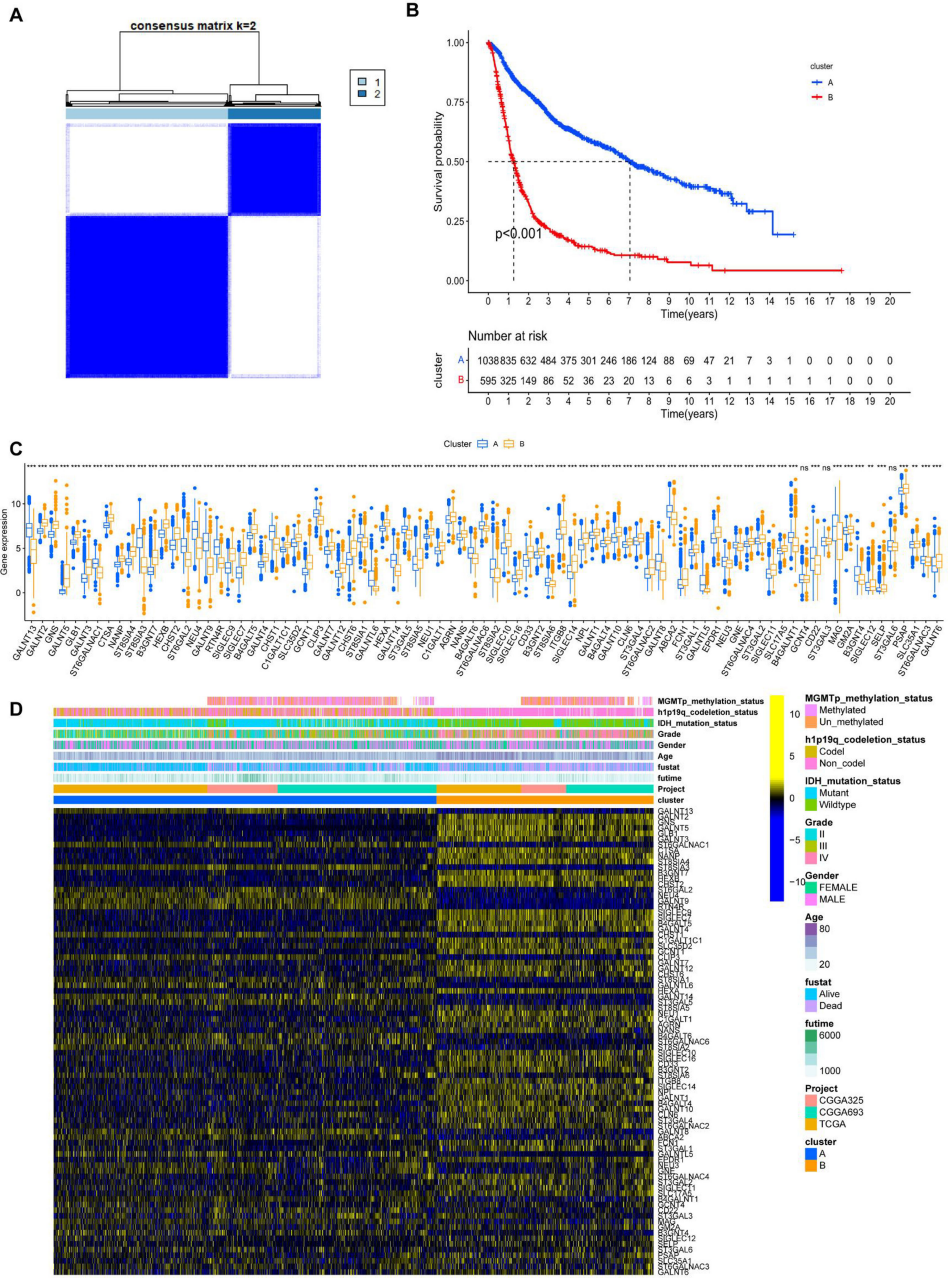
Unsupervised hierarchical clustering analysis of mRNA sequencing data from patients with LGG and GBM based on sialylation-related genes. **(A)** Unsupervised clustering of patients with LGG and GBM. **(B)** Prognostic analysis of patients in Clusters-sia A and B. Significance was calculated with the log-rank test. **(C)** The panel of sialylation-related genes exhibits differential gene expression levels between clusters Clusters-sia A and B. **(D)** Correlation analysis between the differentially expressed genes within the sialylation-related gene panel and clinical phenotypes. ***P < 0.001.

Consensus clustering was performed on 1,688 GBM/LGG samples based on 83 sialylation-related genes, with cluster numbers (k) ranging from 2 to 9. Analysis of cumulative distribution function (CDF) curves derived from the consensus score matrix identified k=2 as the optimal cluster number. (**Figure 1A**, **Supplementary Figure S2**). To further explore the potential implications of consensus cluster analysis for GBM/LGG patients, we analyzed the survival rates between the two groups, as illustrated in **Figure 1B**. Clusters-sia A (consisting of 1067 cases) and Clusters-sia B (consisting of 611 cases) displayed significant variations in survival rates, representing the good and poor outcome groups, respectively (**Figure 1B**). We also investigated the expression levels of the sialylation-related genes in the two groups, genes including GALNT5 (logFC=1.9), SIGLEC7 (logFC=1.5) and SIGLEC9 (logFC=1.5) were upregulated, genes including ST8SIA3(logFC=-3), GALNT9 (logFC=-2.6) and GALNT13 (logFC=-2.3) were downregulated and GCNT4, ST3GAL3 and GALNT11 showed no variation in group B vs group A. (**Figure 1C**). As illustrated in **Figure 1D**, Clusters-sia B was positively correlated with 1p19q non-codeletion, wild-type IDH1, and shorter survival.

### GSVA, immune infiltration analysis and differential genes enrichment analysis between cluster A and cluster B

**Figure 2:**
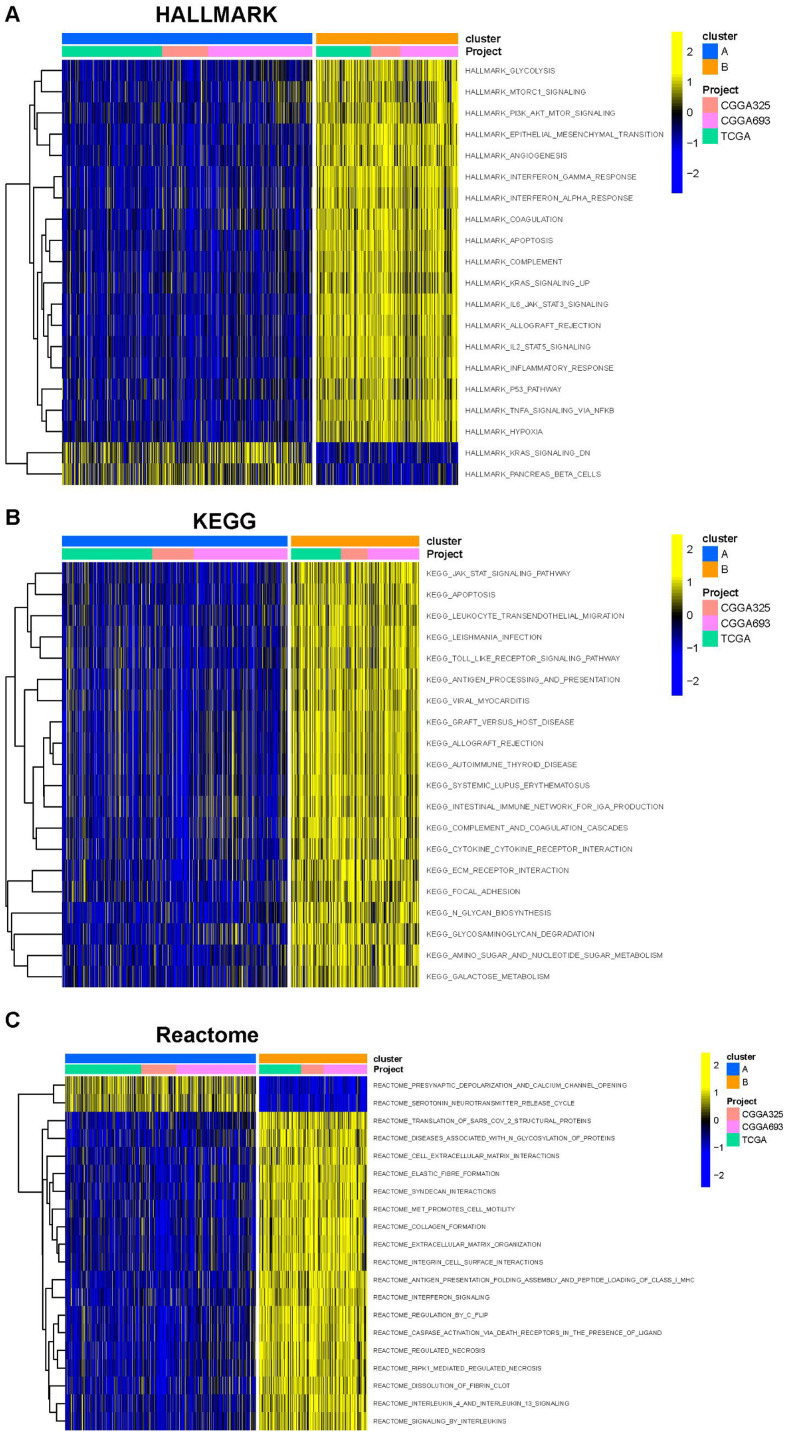
Gene set variation analysis (GSVA) based on the HALLMARK gene set **(A)**, KEGG gene set **(B)**, and Reactome gene set **(C)**.

Gene set variation analysis (GSVA) was performed to screen for biological differences between Clusters-sia A and B. As shown in **Figures 2A, B**, when the KEGG and HALLMARK gene sets were used as reference sets, GSVA enrichment analysis demonstrated that inflammatory and immune pathways were enriched in cluster B, including INTERFERON_GAMMA_RESPONSE, INTERFERON_ALPHA_RESPONSE, IL6_JAK_STAT3_SIGNALING, TNFA_SIGNALING_VIA_NFKB, and TGF_BETA_SIGNALING. DNA replication (consisting of E2F_TARGETS) and cell cycle-associated pathways (consisting of G2M_CHECKPOINT and MYC_TARGETS_V1) were also enriched in cluster 2. Furthermore, the ANGIOGENESIS pathway, which is highly associated with tumor growth and metastasis, was enriched in Clusters-sia B. Inflammatory pathways, such as INTERLEUKIN_4_AND_INTERLEUKIN_13_SIGNALING and SIGNALING_BY_INTERLEUKINS, were enriched in Clusters-sia B based on the Reactome gene set (**Figure 2C**).

**Figure 3:**
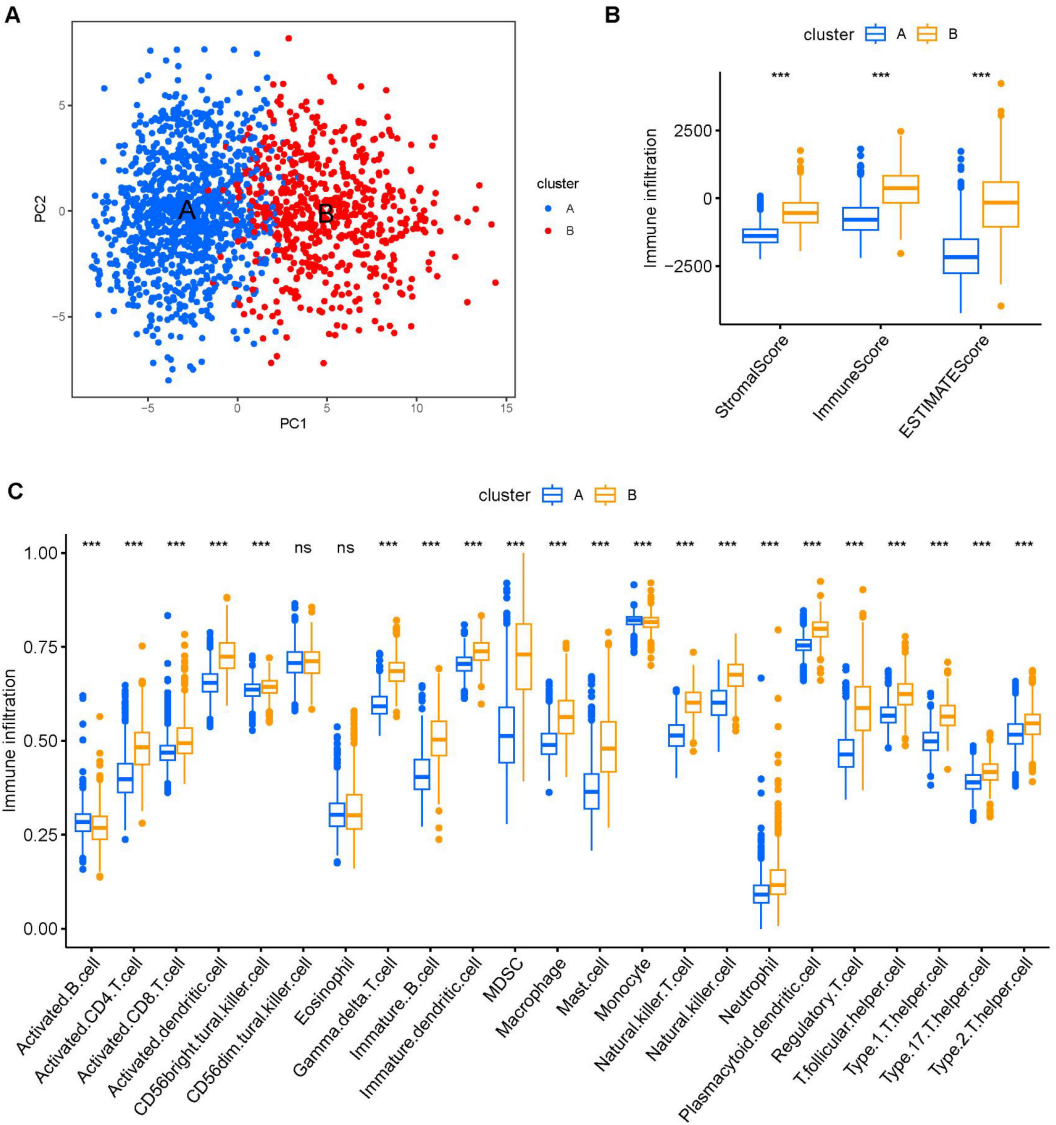
The composition of tumor immune microenvironment cells in Clusters A and B. **(A)** Scatter plot of the principal component scores in the validation set (using the weights derived from the discovery set) demonstrating the distinction between clusters A and B. **(B)** The distribution of immune score, as inferred by ESTIMATE algorithm, between the two clusters in the cohort ***P < 0.001. **(C)** The infiltration abundance of 23 immune cell subsets in the two clusters. ***P < 0.001.

The PCA demonstrated that PC1 accounted for 24% of the dataset variance, whereas PC1 plus PC2 had a 8% proportion of variance. PC1 and PC2 together account for approximately 32% of the dataset. The ESTIMATE algorithm was applied to assess the tumor microenvironment (TME) between Clusters A and B. There were notable differences in immune cell infiltration between the two consensus clusters (A and B), with Cluster B exhibiting significantly higher overall infiltration levels compared to Cluster A. (**Figures 3A, B**). As a result, we classified Cluster A as “immune-cold” tumors and Cluster B as “immune-hot” tumors.

**Figure 4:**
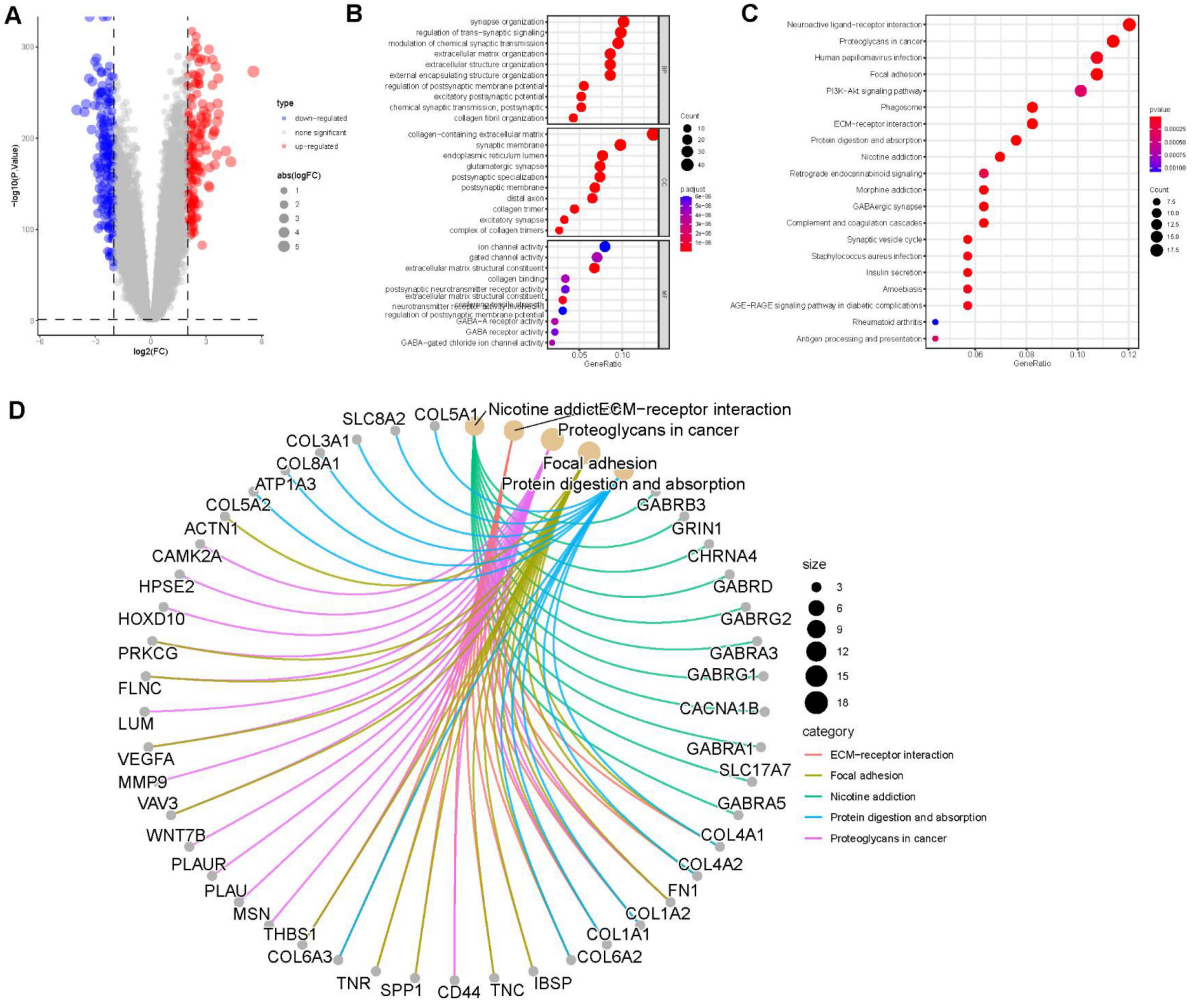
Enrichment analysis. **(A)** Volcano plot demonstrating differentially expressed genes (DEGs) between the two clusters. **(B)** Gene Ontology (GO) enrichment analysis of DEGs, including biological process (BP), cellular component (CC), and molecular function (MF) categories. **(C)** Top 20 enriched pathways from KEGG analysis of DEGs. **(D)** Correlation between DEGs and the top 5 enriched KEGG pathways.

A total of 352 differentially expressed genes (DEGs) (|logFC| > 2, P < 0.05) were identified between the two clusters (**Figure 4A**). Gene Ontology (GO) analysis revealed that the DEGs were significantly enriched in extracellular matrix organization within the Biological Process (BP) category, collagen-containing extracellular matrix and endoplasmic reticulum lumen in the Cellular Component (CC) category, and collagen binding in the Molecular Function (MF) category (**Figure 4B**). KEGG pathway analysis indicated that the DEGs were primarily enriched in the ECM-receptor interaction and TGF-beta signaling pathways (**Figure 4C**). **Figure 4D** illustrates the associations between the DEGs and the top five enriched KEGG pathways.

**Figure 5:**
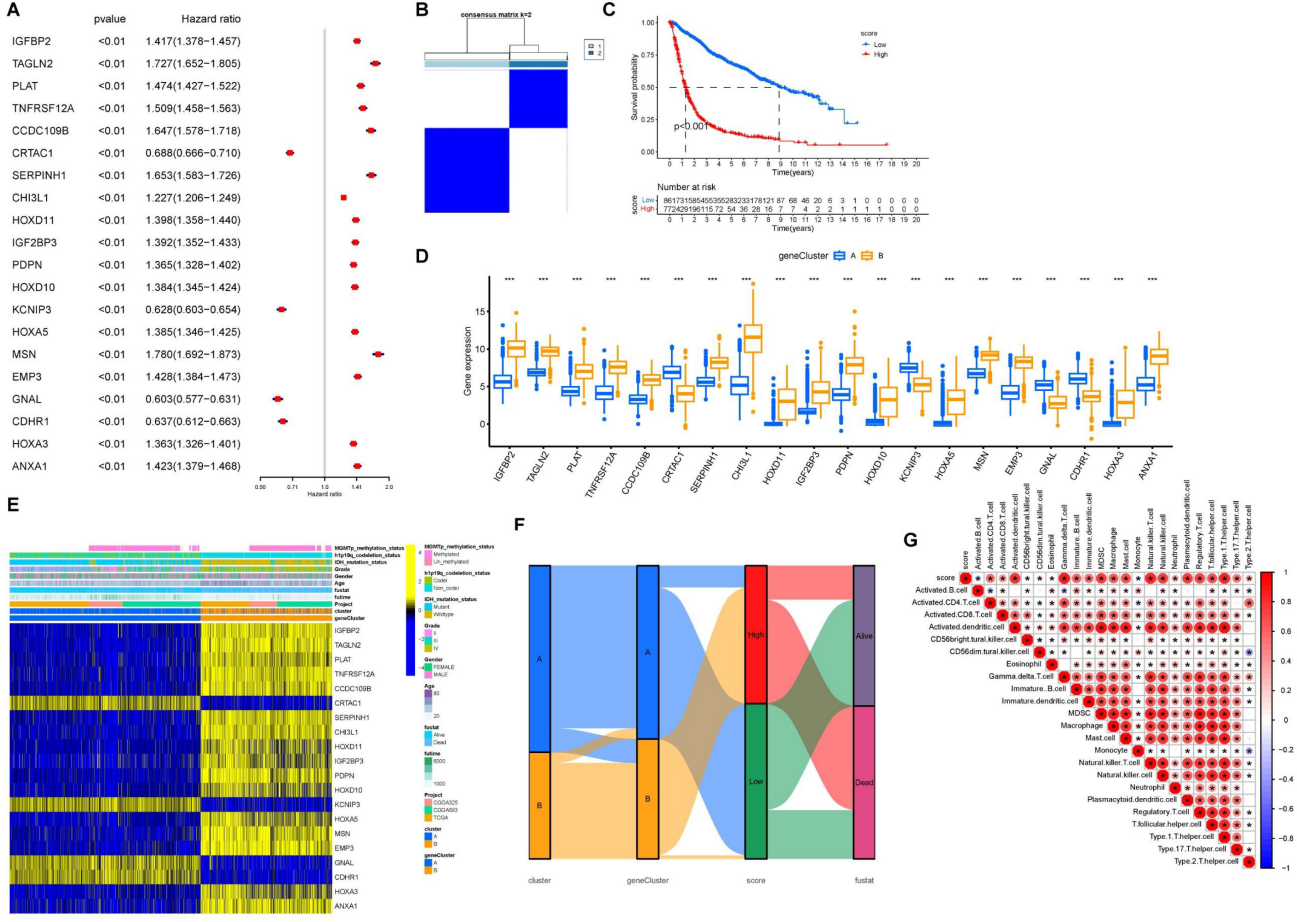
Cohort group based on PCA score. **(A)** Forest plot of HR and P value for the 20 model genes. **(B)** Grouping of GBM/LGG cohort patients into two molecular clusters (k = 2) based on the 20 model genes. **(C)** OS in low and high PCA scores. **(D)** Levels of the 20 model genes expressed between gene clusters A and B. **(E)** Heatmap of the 20 model genes and clinical factors. **(F)** Alluvial diagram illustrating the interrelationship between the two clusters, molecular clusters, clinical status, and survival status in the GBM/LGG corhort. **(G)** Correlation matrix of PCA score with infiltrating immune cells in the tumor microenvironment. *P<0.05.

Univariate Cox regression analysis was conducted on the 352 differentially expressed genes. **Figure 5A** shows the top 20 genes selected for further analysis and ranked by p-values. Based on these 20 genes, an unsupervised clustering analysis was conducted and a two-cluster solution was found to be the most suitable (**Figure 5B**). Additionally, the expression levels of these 20 genes varied significantly between the clusters (**Figure 5D**). The expression levels of CRTAC1, KCNIP3, GNAL, and CDHR1 were higher in Clusters-20 B than in cluster A, and the hazard ratios (HR) for all four genes were <1, suggesting that these genes may play important protective roles. The distribution of clinical features and the expression of prognostic genes across the two gene clusters are shown in **Figure 5E**. Principal component analysis (PCA) was conducted based on the expression profiles of the selected 20 genes. The composite score was calculated as the difference between the first and second principal components (PC1 and PC2): Patients were then stratified into high- and low-score groups for survival analysis, which showed that higher scores were associated with a poorer prognosis (**Figure 5C**). The PCA scores were significantly higher in both Cluster A and Clusters-sia A, suggesting a potential association between the underlying molecular characteristics of these subtypes (**Figure 5F**). The correlation between PCA score and immune cell infiltration indicated that the PCA score was positively associated with immune cell infiltration (**Figure 5G**). The correlation between PCA score and clinical information was shown in **Supplementary Figure S3**.

### Genetic alteration of prognostic genes

**Figure 6:**
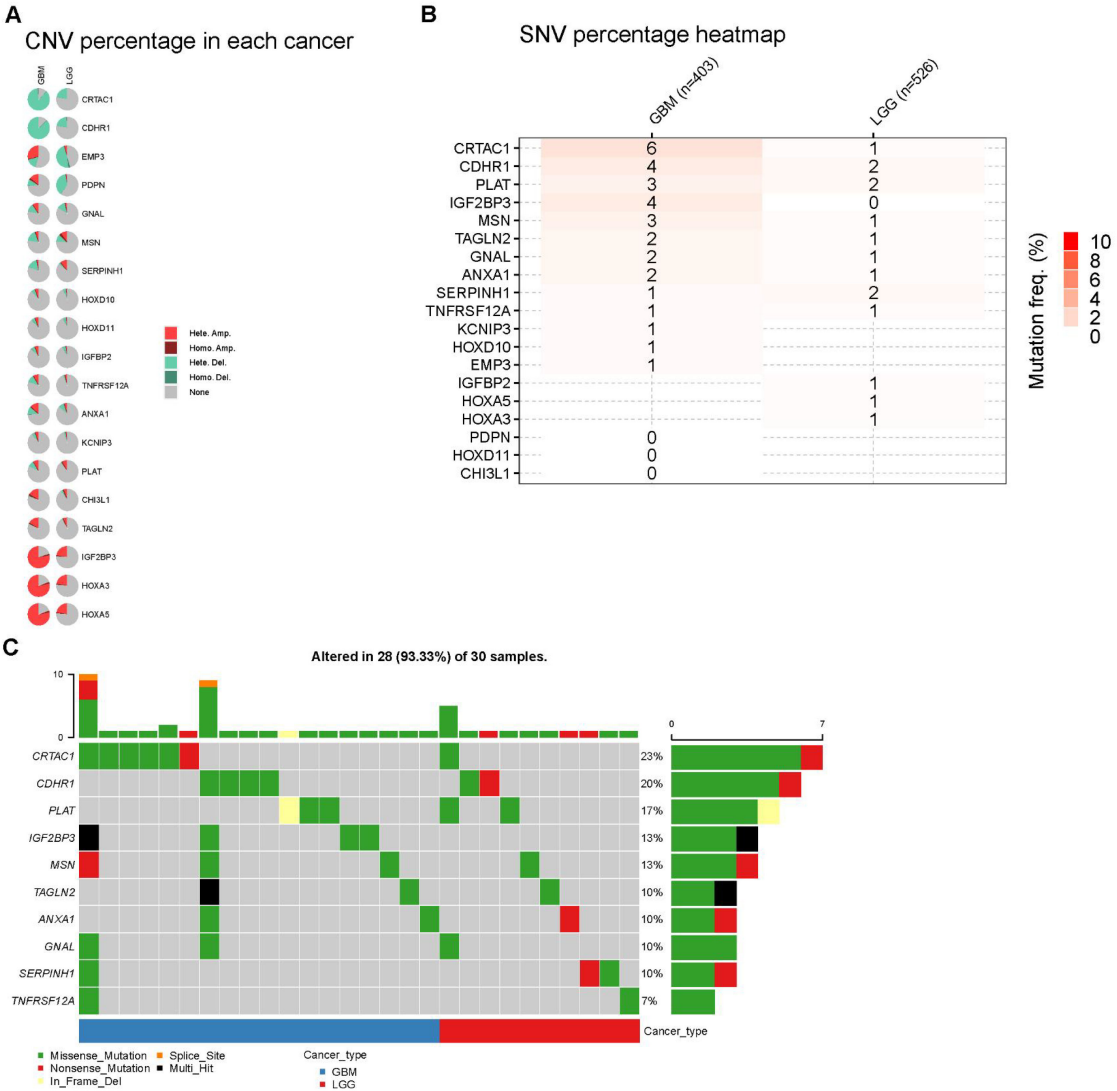
Landscape of genes in GBM and LGG cohort. **(A)** Pie plot summarizes the Copy number variation (CNV) of selected prognostic genes **(B, C)** Diagram of SNV and somatic mutations for the prognostic genes.

We further examined the genetic alterations in the 20 prognostic genes. A pie chart illustrates the copy number variations (CNVs) of these genes in the GBM/LGG cohort, including heterozygous deletion, heterozygous amplification, homozygous deletion, and homozygous amplification (**Figure 6A**). CDHR1 and CRTAC1 exhibited the highest frequencies of CNV loss, whereas HOXA5 and HOXA3 showed significant CNV amplification in patients with GBM. We examined the single-nucleotide variations (SNVs) of these genes. Overall, the SNV frequencies were relatively low, with CRTAC1 showing the highest SNV frequency in patients (**Figures 6B, C**).

**Figure 7:**
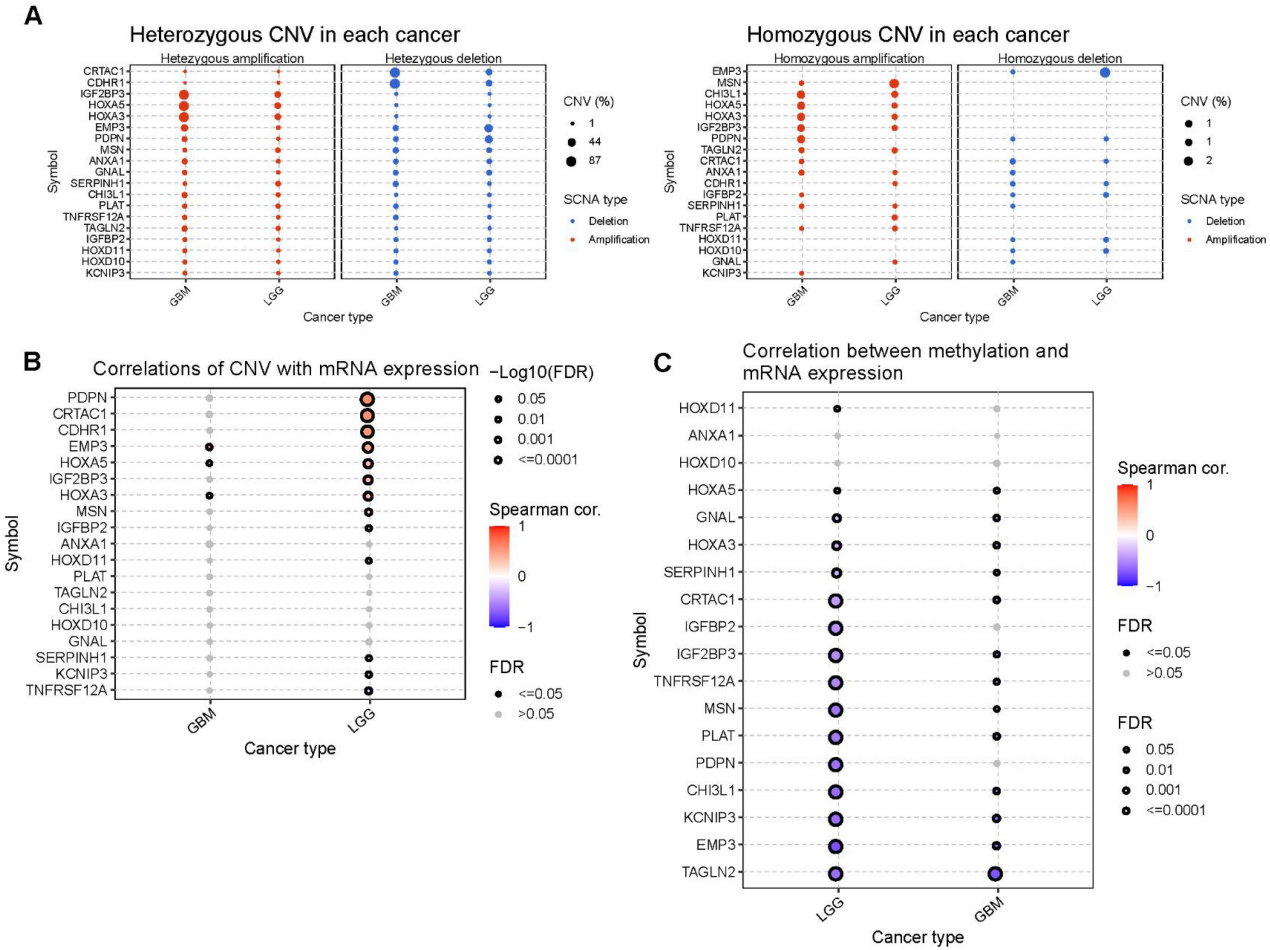
The CNV and methylation of prognostic genes in GBM and LGG cohort. **(A)** Heterozygous and homozygous CNV of prognostic genes. **(B)** Correlation between CNV and mRNA expression. **(C)** Correlation between methylation level and mRNA expression.

A significant positive correlation was observed between the CNV levels and mRNA expression of PDPN, CDHR1, and CRTAC1 in patients with LGG (**Figure 7B**). Finally, we investigated the differences in DNA methylation in the GBM/LGG cohort (**Figure 7C**) and found that gene expression levels were generally negatively correlated with DNA methylation (**Figure 7C**).

### Higher PCA score correlates with increased immune cell infiltration, elevated immune checkpoint expression, enhanced chemokine expression, and improved immunotherapy efficacy

**Figure 8:**
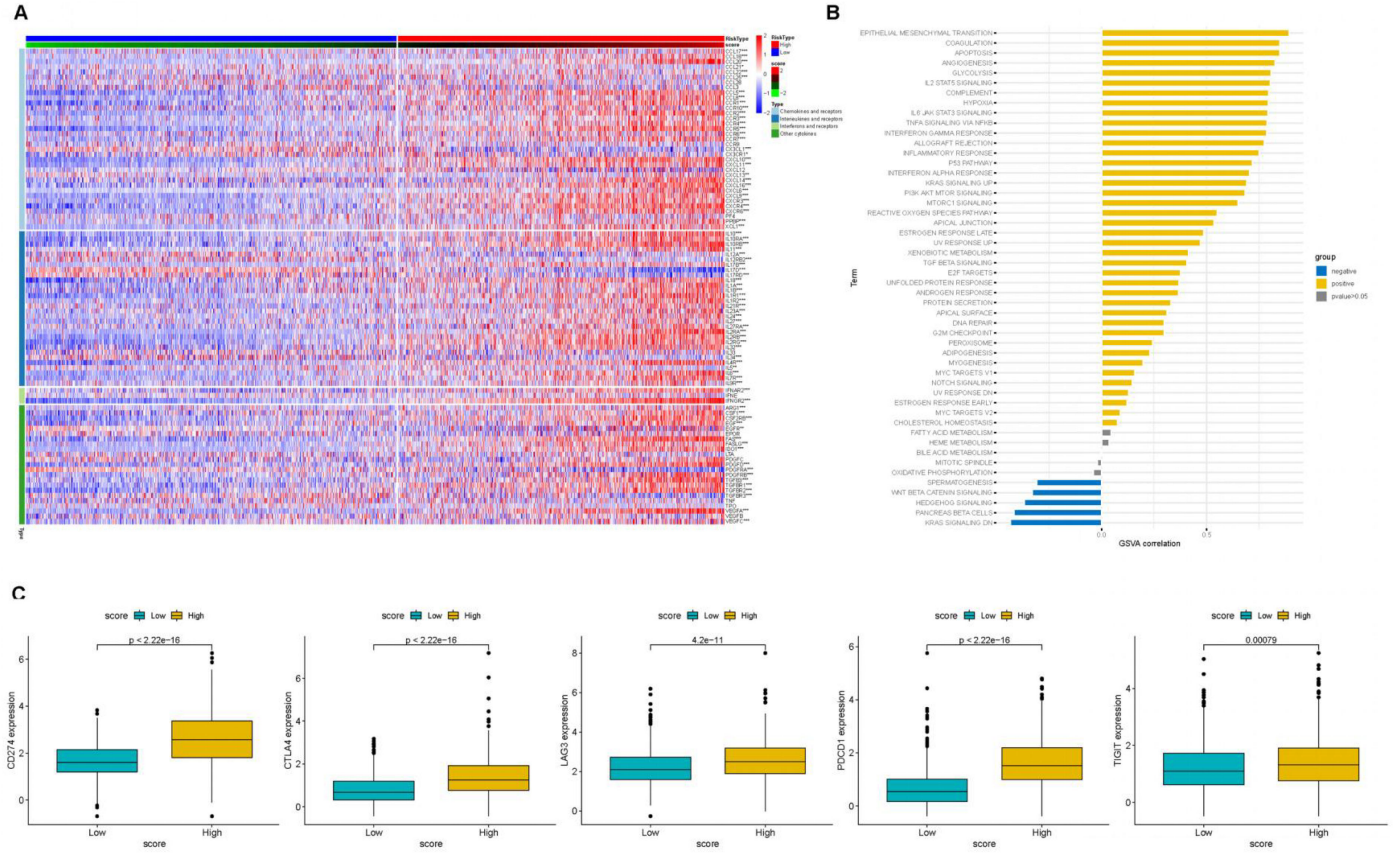
The association of PCA scores with immune-related genes. **(A)** Heatmap of Chemokine Gene Expression and PCA Score. Rows represent genes of chemokines and receptors, and columns represent samples expressed in the high- and low-PCA score subgroups. **(B)** The correlation of PCA score with HALLMARK pathway scores. **(C)** ICB-relevant genes (CD274, CTLA4, LAG3, PDCD1, and TIGIT 2) expressed in the high- and low-PCA score subgroups.

**Figure 9:**
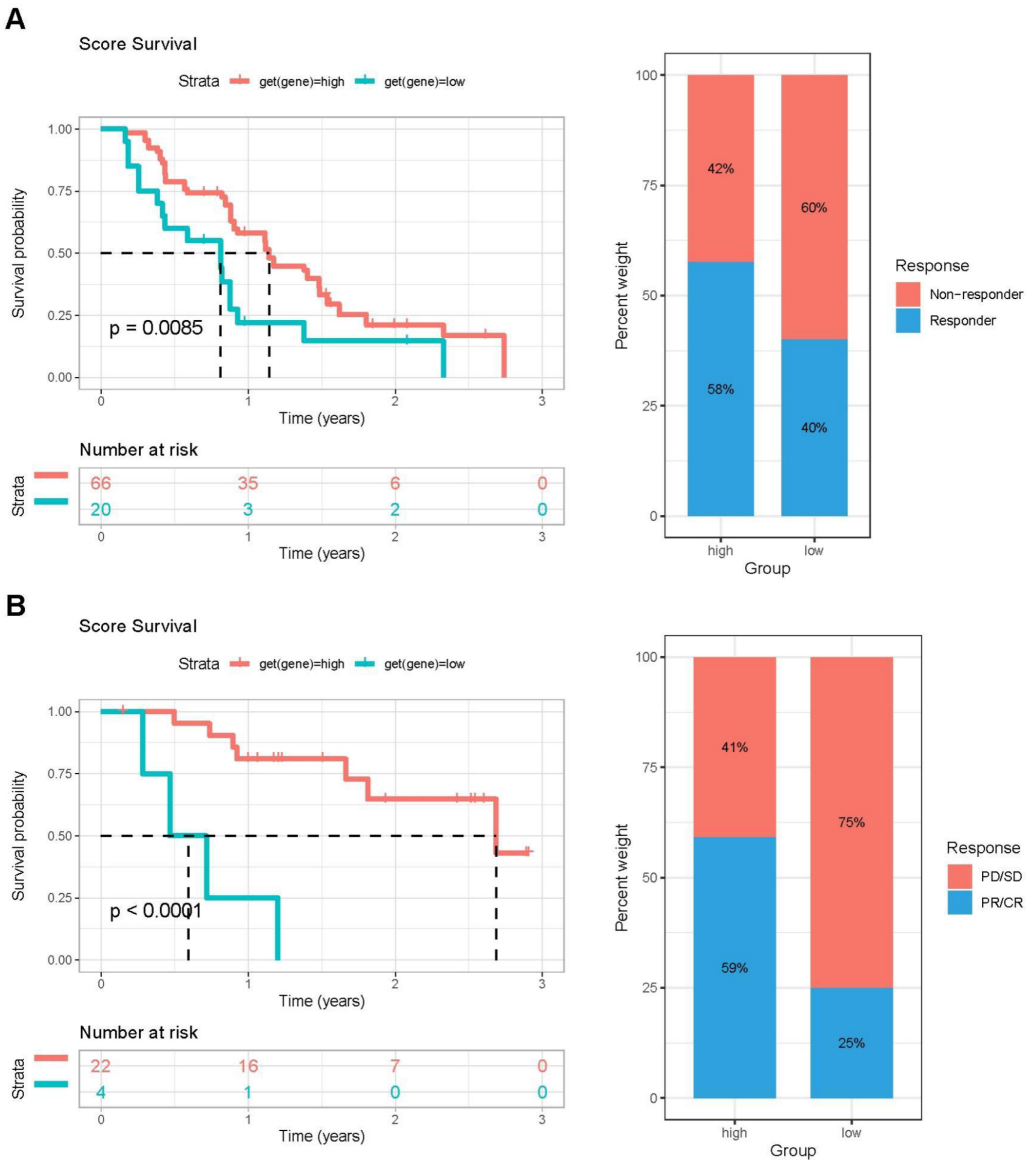
The validation of PCA score effectiveness in immune therapy using two immunotherapy cohorts. **(A)** Left: Kaplan–Meier OS survival curves comparing high- and low-PCA score groups in the GSE61676 cohort. Right: Distribution of patient response status following ICI treatment between high- and low-PCA score groups in the GSE61676 cohort. **(B)** Left: Kaplan–Meier OS survival analysis of patients stratified by PCA score (high vs. low) in the IMvigor210 cohort. Right: Proportion of patients with different clinical outcomes after ICI treatment in high- and low-PCA score groups in the IMvigor210 cohort.

To further investigate the impact of PCA score-based grouping on tumor microenvironment classification and immunotherapy efficacy, we first examined the expression relationships of chemokines and their receptors between the high and low PCA score groups (**Figure 8A**). The results showed that Chemokines and their receptors were predominantly expressed in the high-score group. These results indicated that patients with high PCA scores may be more sensitive to immunotherapy. Importantly, tumor inflammatory pathways, including IL2-STAT5 signaling, IL6-JAK-STAT3 signaling, and interferon-alpha response, were predominantly enriched in the high PCA score group (**Figure 8B**). Furthermore, the expression levels of immune checkpoint genes (CTLA4, CD274, PDCD1, TIGIT, and LAG3) were higher in the high-scoring group compared to the low-scoring group (**Figure 8C**). These findings support our hypothesis that patients with tumors exhibiting high PCA scores may be more responsive to immunotherapy. To validate this hypothesis, we conducted additional analyses using immunotherapy datasets and demonstrated that patients with high PCA scores showed greater sensitivity to ICI treatment in the GSE61676 dataset (**Figures 9A, B**).

### KCNIP3 inhibited cell proliferation, colony formation and metastasis *in vitro* and *in vivo*

**Figure 10:**
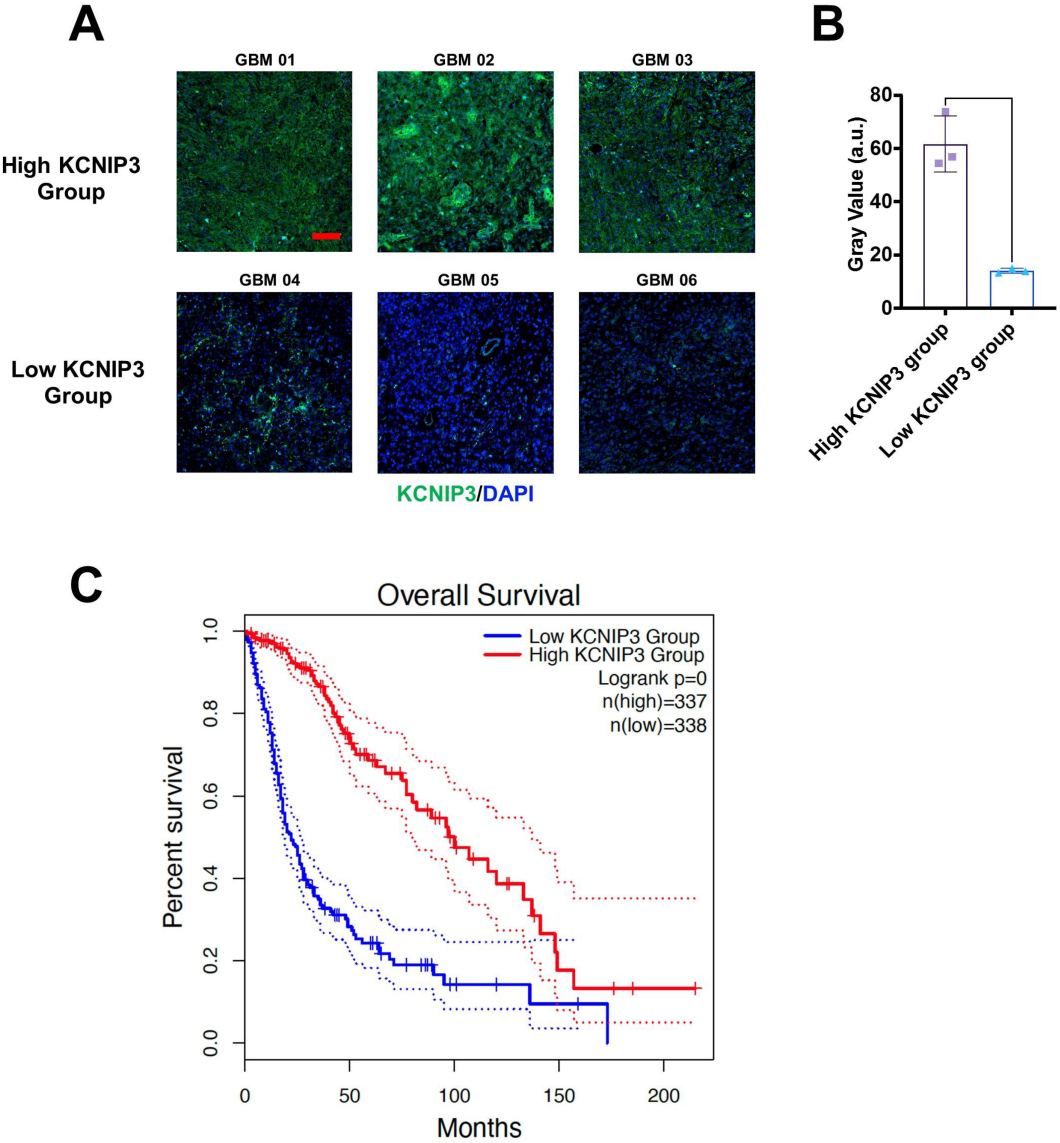
Differential expression and prognosis of KCNIP3 in tumor tissues in patients with glioblastoma. **(A)** Representative images of immunohistochemistry staining for detecting KCNIP3 expression in GBM tissues. Green fluorescence indicates KCNIP3, and DAPI labels cell nuclei. Scale bar: 100 μm. **(B)** KCNIP3 staining was semi-quantitatively evaluated by gray value. **(C)** Survival curves of patients with LGG and GBM with low- or high-KCNIP3 expression, sourced from the TCGA database.

To further investigate the roles of these 20 genes in the proliferation and invasion of LGG and GBM tumor cells, we focused on genes with a hazard ratio (HR) less than 1. We first validated the expression of these four genes and their association with patient survival in brain tumor samples using immunohistochemistry. The results showed that Patients with high KCNIP3 expression had longer survival times (**Figure 10**).

**Figure 11:**
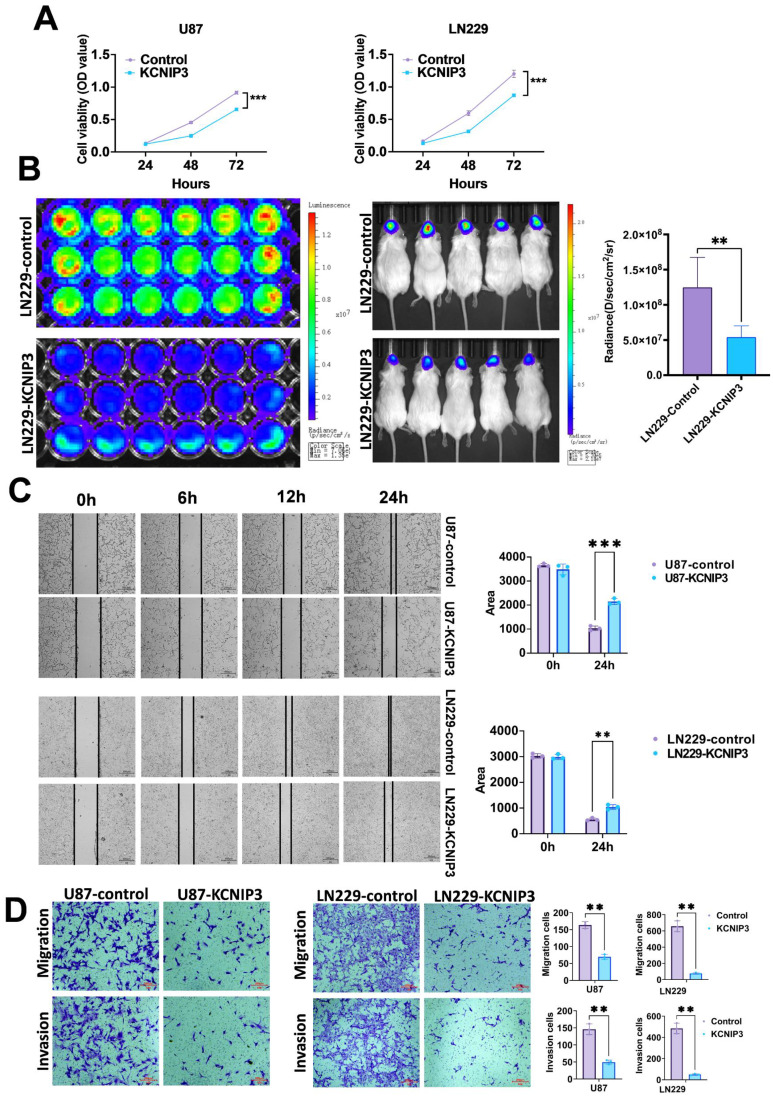
The proliferation, migration, and invasion of GBM in U87 and LN229 cells are inhibited by KCNIP3 overexpression both *in vitro* and *in vivo*. **(A)** Graphs showing the results of CCK-8 assays measuring the viability of U87, U87- KCNIP3, LN229 and LN229- KCNIP3 cells for 48 and 72 h. Cell viability is expressed as the percentage of OD450 in the transfected cells relative to U87 or LN229 at each time point. **(B)** The IVIS Imaging System was used to assess the *in vivo* proliferation of orthotopic xenografted LN229-KCNIP3 in mice (n = 5). **(C)** Wound-healing assays demonstrated the migration ability of U87 and LN229 cell lines with KCNIP3 overexpression and its quantification. Scale bar: 500 μm。**(D)** Transwell assays demonstrated the migration and invasion abilities of the U87 and LN229 cell lines with KCNIP3 overexpression and their quantification. **P < 0.01, ***P < 0.001.

We overexpressed KCNIP3 in U87 and LN229 cells using lentiviral vectors. After selecting the positive cell clones, we successfully established U87 and LN229 cell lines overexpressing KCNIP3. The effect of KCNIP3 on cell proliferation was assessed using the CCK-8 assay (**Figure 11A**). The results showed that at 48 and 72 h post-seeding, the proliferation rates of U87 and LN229 cells overexpressing KCNIP3 were significantly lower than those of control cells. More importantly, an intracranial xenograft model was established using U87 cells co-expressing KCNIP3 and firefly luciferase, which showed significantly slower growth than control U87 cells (**Figure 11B**). Wound healing and Transwell assays indicated that KCNIP3 inhibited cell invasion (**Figures 11C, D**). Collectively, these results suggest that KCNIP3 suppresses tumor cell proliferation and invasion both *in vitro* and *in vivo*.

## Discussion

In this study, we first performed unsupervised clustering of a combined GBM and LGG cohort based on a sialylation-related gene set and identified two distinct molecular subtypes. We then conducted a univariate Cox regression analysis on differentially expressed genes (DEGs) between the two clusters and selected 20 genes with p-values < 0.05. Using these 20 genes, we divided the cohort into two groups and performed PCA, defining the PCA score as PC1 minus PC2. A higher PCA score was associated with poorer survival, increased expression of immune checkpoint genes, elevated levels of chemokines, and a potentially improved response to immune checkpoint inhibitor (ICI) therapy. In glioma, the subtype without mutations in the IDH gene is typically highly aggressive, associated with a poor prognosis, and prone to recurrence. Consistent with this, our high-risk patient group was characterized by a high frequency of 1p/19q non-codeletion and IDH wild-type status, which aligns with their unfavorable clinical outcomes.

Sialylation is a common modification of glycoproteins and glycolipids on the surface of cancer cells that shows significant changes in various cancers. Studies have shown that Increased sialylation is closely associated with tumor invasiveness, progression, and metastasis (26). In lung cancer, alterations in the glycosylation patterns of proteins on the cell surface are considered important factors influencing interactions in the tumor microenvironment, potentially promoting tumor metastasis and liver colonization (27). In breast cancer, sialylation promotes the pathogenicity of cancer cells by regulating gene expression signatures, which may involve glycoproteins and glycolipids (28). In ovarian cancer, changes in sialylation are important markers of cancer progression, potentially affecting the survival, proliferation, and metastatic capabilities of cancer cells (29). In pancreatic cancer cells, overexpression of sialic acid can induce immunomodulatory effects by binding to siglec-receptors, a mechanism that may promote tumor progression by influencing macrophage differentiation (30). In prostate cancer, sialylation promotes tumor growth and invasion and inhibits the cytotoxic effects of immune cells by binding to Siglec-7 and Siglec-9 (31). In addition, abnormal sialylation is associated with multidrug resistance in cancer. In acute myeloid leukemia, changes in sialylation may influence cell resistance (32) by regulating the PI3K/Akt signaling pathway and related protein expression. These studies indicate that sialylation plays a crucial role in the progression and metastasis of various cancers, suggesting that treatments targeting the sialylation pathway may offer new strategies for cancer therapy. Excessive sialylation can directly affect tumor growth, metastasis, immune evasion, and drug resistance. For instance, overexpression of ST3GAL4 has been associated with poor prognosis and disease progression in gastric, pancreatic, cervical, and uterine cancers (14, 33, 34). In gliomas, ST6GAL1 promotes GBM growth through α2,6-sialylation (35). GBM cells utilize excessive sialylation to evade immune surveillance and targeting the siglec-sialic acid axis can suppress tumor growth in mouse models, especially when combined with an immune checkpoint blockade (36). These data provide a strong rationale for using sialylation-related genes to predict patient outcomes and responses to ICIs.

Following sialylation-based clustering, immune cell infiltration was found to be significantly higher in Cluster-sia B than in A, suggesting a link between sialylation and the tumor immune microenvironment. Although significant progress has been made significant progress, its clinical application remains limited owing to its low response rates, side effects, and resistance (37, 38). Our study suggests that sialylation gene signatures can predict ICI efficacy.

Our results showed that Cluster-20 A contained more cases with IDH mutations and a higher frequency of 1p/19q codeletion than Cluster-20 B, indicating a better prognosis. Similarly, **Figure 6C** demonstrates that Cluster-20 A had significantly better survival than Cluster-20 B, consistent with its molecular features. High PCA scores were associated with worse outcomes, likely reflecting distinct tumor immune landscapes. Notably, tumors with high scores showed a reduced correlation with antitumor immune cells such as activated B cells, CD4 + T cells, and CD8 + T cells. Moreover, higher PCA scores correlated with the upregulation of immune checkpoint genes (CD274, CTLA4, LAG3, PDCD1, and TIGIT), indicating a more immunosuppressive microenvironment and increased chemokine expression. This finding suggests that patients with high PCA scores may benefit more from ICI therapy, a hypothesis supported by validation in melanoma and urothelial carcinoma cohorts.

The seemingly paradoxical association between high PCA scores, worse prognosis, and enhanced ICI response originates from the “immune hot yet aggressive” TME in high-score patients, driven by three interconnected mechanisms: First, our GSVA and immune infiltration analyses confirm high PCA scores correlate with concurrent enrichment of immune-related pathways and increased immune cell infiltration, alongside pro-tumorigenic pathways; this co-activation means the “immune hot” TME coexists with aggressive tumor traits that worsen baseline prognosis but provide abundant immune cells primed for ICI response. Second, high PCA scores are associated with upregulated immune checkpoint genes, where high immune cell abundance induces chronic antigen stimulation and “immune exhaustion”—impairing anti-tumor immunity to drive poor prognosis, but this exhaustion is reversible via ICI (restoring cytotoxic T cell function) to improve response. Third, high PCA scores link to adverse molecular subtypes (1p19q non-codeletion, IDH1 wild-type) that amplify poor survival via genomic instability and standard therapy resistance, yet do not abolish the “immune hot” TME, preserving ICI responsiveness. Collectively, high PCA score patients exhibit a TME with conflicting features: abundant immune infiltration paired with aggressive tumor behavior and immune exhaustion—a balance that explains the paradox and underscores the PCA score’s value in identifying patients who may benefit from ICI despite poor baseline outcomes.

The coexistence of enhanced tumor invasiveness and improved ICI suitability in high PCA score patients stems from abnormal sialylation’s dual, context-dependent roles: on one hand, high PCA scores reflect tumor cell hyper-sialylation, which directly drives invasiveness by modifying cell adhesion molecules and matrix metalloproteinases to reduce cell-cell adhesion and promote extracellular matrix degradation—further reinforced by enrichment of pro-invasiveness pathways and pro-tumorigenic pathways in high-score groups; on the other hand, this hyper-sialylation enhances ICI suitability by remodeling the TME into an “immune-responsive” state: it recruits immune cells via upregulated chemokines and pro-inflammatory cytokines, and while it initially promotes immune evasion, it also induces chronic antigen stimulation that upregulates immune checkpoints—creating “reversible immune exhaustion” where abundant infiltrating T cells, though functionally suppressed, retain cytotoxic potential to respond to ICI. This is not a contradiction but reflects sialylation as a “double-edged regulator”: high PCA scores capture this dual state, explaining why high-score patients show better ICI response in our GSE61676 validation cohort despite worse baseline invasiveness.

The essence of an “immune-hot” TME lies in the balance between immune activation and immunosuppression. A true “immune-hot” or “immune-inflammatory” TME is characterized not merely by the presence of effector immune cells (e.g., cytotoxic T cells, NK cells) but by active immune responses. Any effective immune response is accompanied by intrinsic negative feedback regulatory mechanisms to prevent excessive immune damage. As key immunosuppressive components, Treg cells are often recruited and activated synchronously with effector T cell infiltration. Thus, the enrichment of Treg cells observed in Cluster B is a normal manifestation of such active immune responses coupled with immunosuppression.

The KCNIP gene family (KCNIP1-4) belongs to the calcium sensor protein family and consists of four EF-hand calcium-binding domains. The EF-hand domain is a calcium-binding domain that plays crucial roles in many cellular processes. EF-hand proteins are typically divided into two categories: calcium sensors and buffers. Calcium sensors translate calcium signals into various cellular responses, while calcium buffers regulate the levels of free calcium ions in the cytoplasm (39). In neurons, EF-hand calcium sensor proteins, such as calmodulin (CaM) and STIM, play crucial roles in the cytoplasm and endoplasmic reticulum. CaM is a versatile cytoplasmic calcium sensor that interacts with structurally diverse target proteins and regulates their functions. In contrast, STIM is specifically responsible for calcium sensing in the endoplasmic reticulum and self-regulates the stability of its calcium-sensing domain through its EF-hand structure (40). In addition, EF-hand calcium-binding proteins play a crucial role in neuronal calcium signaling (41). To date, no study has reported the role of KCNIP3 in GBM. However, previous work suggests its involvement in emotional regulation (42) and anti-tumor activity via the Wnt/β-catenin pathway inhibition in papillary thyroid carcinoma (43).

Functionally, we demonstrated that KCNIP3 overexpression in U87 and LN229 glioma cells significantly inhibited cell proliferation, invasion, and migration. These findings suggested that the sialylation gene signature may serve as a predictor of patient prognosis and immunotherapy efficacy.

This apparent contradiction arises from the context-dependent role of KCNIP3 expression in glioma: In fact, as shown in **Figure 5D**, KCNIP3 expression is higher in Cluster A. In the poor-prognosis Cluster B, KCNIP3 is relatively low but may be moderately upregulated as a compensatory response to counteract excessive pro-tumorigenic signaling. However, even with this partial upregulation, KCNIP3 expression in Cluster B remains insufficient to fully reverse the aggressive phenotype of the cluster—explaining why Cluster B still has worse prognosis overall. Meanwhile, Cox regression analysis confirms KCNIP3 itself acts as a protective factor (HR < 1), meaning higher KCNIP3 expression within any given cluster correlates with better survival; this is further supported by **Figure 10C**, where KCNIP3 overexpression in glioma cells inhibits proliferation.

## Conclusions

This study was based on a retrospective analysis and future validation in a clinical setting is required. Although immunohistochemistry confirmed the differential expression of score-related genes between GBM and precancerous tissues, the sample size was limited. Larger multicenter studies with long-term follow-up are required to validate our findings. Nevertheless, our results suggest that the proposed score may serve as a valuable prognostic biomarker in patients with GBM and LGG. Further studies are warranted to address these limitations and enhance their clinical relevance.

## Data Availability

The original contributions presented in the study are included in the article/**Supplementary Material**. Further inquiries can be directed to the corresponding authors.
