# Supplementary material for: Sialylation-related gene signature predict prognosis and immunotherapy efficacy in low- and high-grade glioma: a PCA-based stratification study
Source: Front Oncol. 2025 Dec 9;15:1676103. doi: 10.3389/fonc.2025.1676103 (PMC12722909; doi:10.3389/fonc.2025.1676103)
Supplement: Supplementary file 1 [file DataSheet1.pdf]

Sialylation-Related Gene Signature Predict Prognosis and Immunotherapy Efficacy in Low- and High-Grade Glioma: A PCA-Based Stratification Study

Supplementary Material:

Supplementary Figure 1-3.

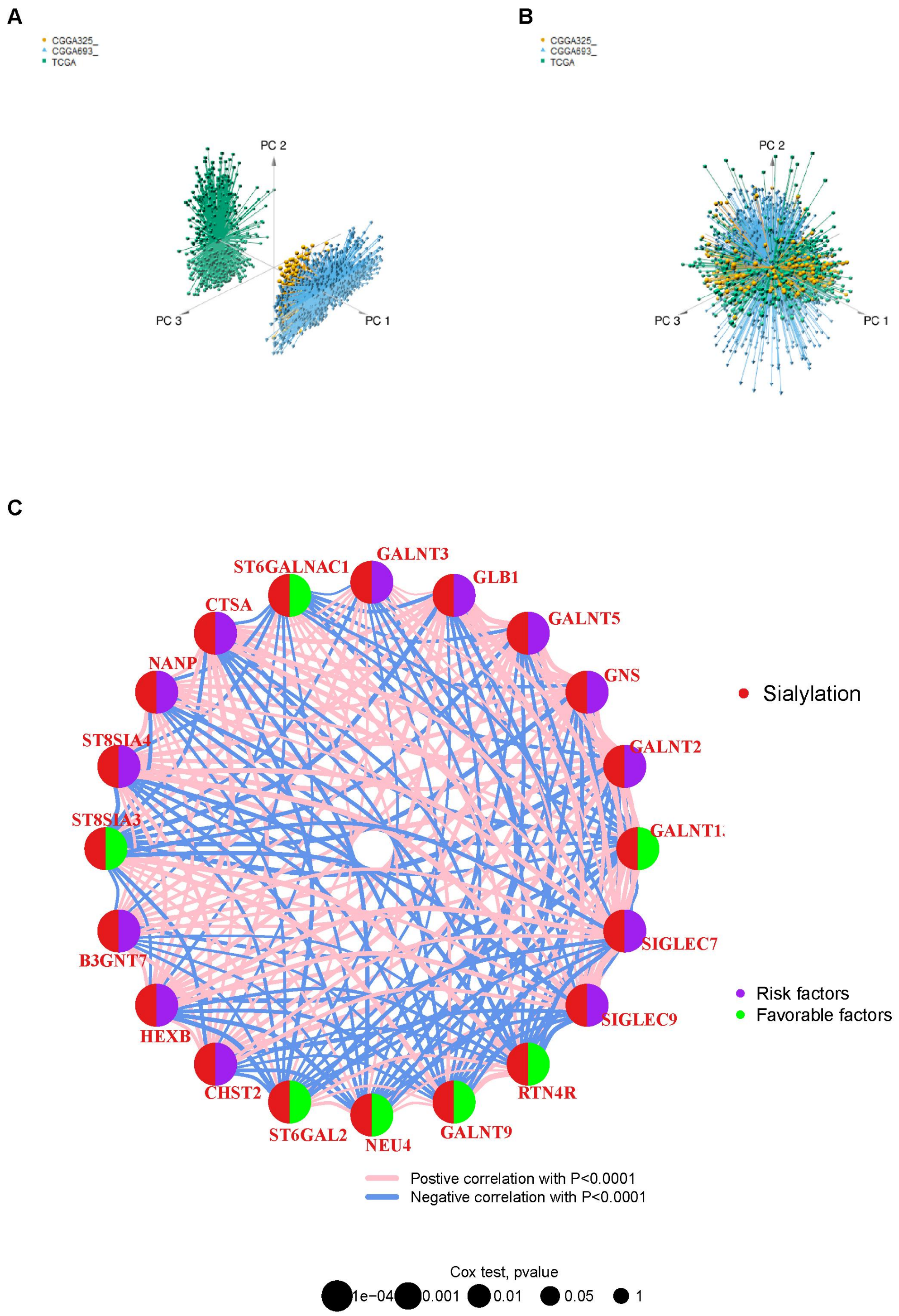

Figure S1. Principal component analysis (PCA) and univariate Cox regression analysis results of sialylation-related genes. PCA showing the distribution of samples before (A) and after (B) merging the TCGA and LGG datasets. (C) Results of univariate Cox regression analysis performed on all sialylation-related genes.

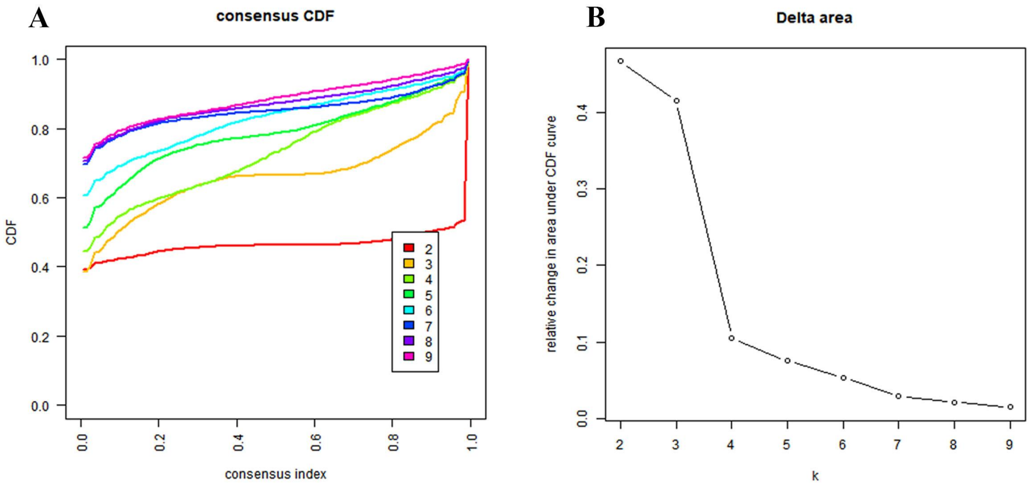

Figure S2. Identification of the sialylation-related genes by LASSO regression. (A) The Cumulative distribution function curves of consensus matrix for each k (indicated by colors). (B) Delta represents the relative change course in the area under the CDF curve when k = 2–9.

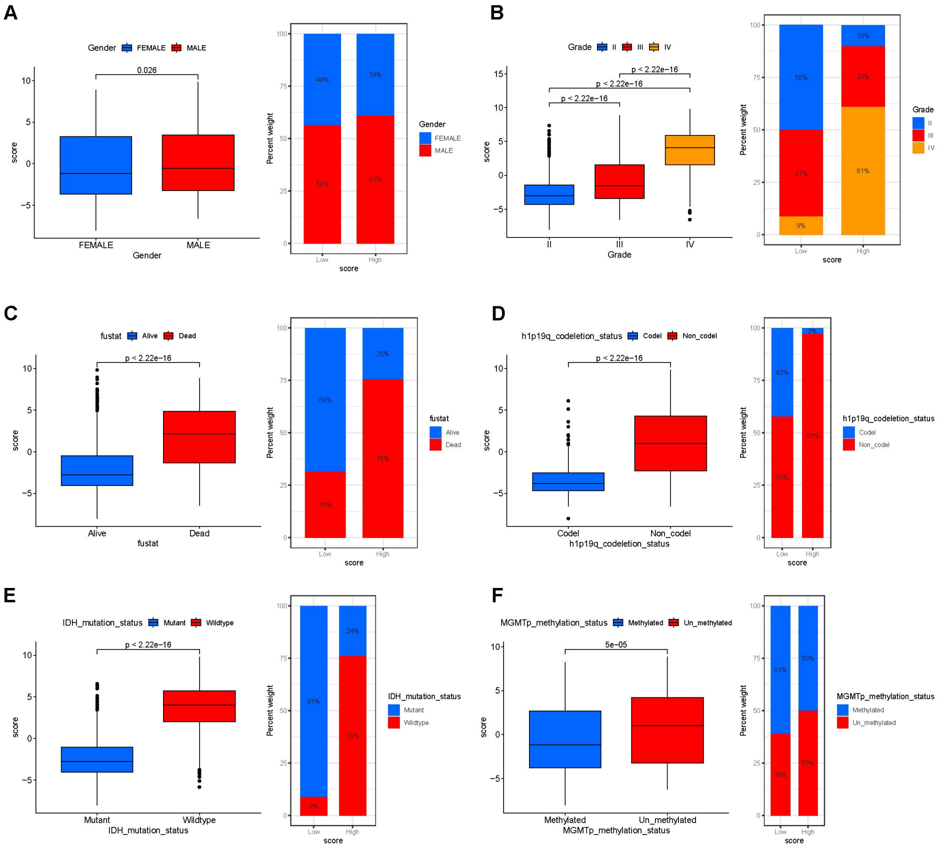

Figure S3. The relationship between the PCA score and clinical characteristics. A. Relationship between age and the PCA score; B. Relationship between tumor grade and the PCA score; C. Relationship between survival status and the PCA score; D–F. Relationships between 1p19q codeletion status, IDH mutation status, and MGMT promoter methylation status and the PCA score.
